# Editorial: Learning and control in robotic systems aided with neural dynamics

**DOI:** 10.3389/fnbot.2023.1193119

**Published:** 2023-04-13

**Authors:** Jiliang Zhang, Long Jin, Tiantai Deng, Muhammad Tahir

**Affiliations:** ^1^College of Information Science and Engineering, Northeastern University, Shenyang, China; ^2^School of Information Science and Engineering, Lanzhou University, Lanzhou, China; ^3^Department of Electronic and Electrical Engineering, The University of Sheffield, Sheffield, United Kingdom; ^4^Department of Electrical Engineering, Lahore University of Management Sciences, Lahore, Pakistan

**Keywords:** neural network, quadratic programming, redundant manipulators, optimal control, neural dynamics, kinematics control, dynamics control, learning and control

Neural network control has been a research hotspot, with broad applied fields in robotics. In recent years, plenty of researchers have devised different types of neural networks, such as zeroing neural networks (ZNN), recurrent neural networks (RNN), and gradient neural networks (GNN) to address complex control issues in robotics in real-life settings. Specifically, different kinds of robotics systems, such as redundant manipulators, multi-agent robotics systems, dual-arm robots, and mobile robots are investigated to achieve different kinds of control features, such as quadratic-programming-based optimal control of redundant manipulators, the remote center of motion control (RCM) of medical robots, and admittance control of robot-environment interaction.

However, some knotty issues still exist, such as uncertain structure parameters, position and orientation control, performance index optimization, and obstacle avoidance.

We envisioned this Research Topic to provide a platform for researchers in this area to publish their latest research ideas. This call received 12 high-quality submissions. After passing through the peer-reviewing process, we have accepted five high-quality papers for publication. A summary of all the accepted papers is given as follows.

In the first paper (Zhu and Tan), a non-linear activation function (NAF) is proposed to construct three recurrent neural networks (RNN) models for sentiment classification. The good accuracy and low loss values brought by the new NAF are validated via Internet Movie Database (IMDB) sentiment classification experiment. A fixed-time convergent recurrent neural network (FTCRNN) model with the NAF is constructed to further solve dynamic problems. The fixed-time convergence property of the FTCRNN model is validated, and the upper bound convergence time of the FTCRNN model is derived as well.

In the second paper (Zhang B. et al.), a fuzzy neural network active motion intention recognition method is proposed to explore the internal connection between the surface electromyogram signal of the human upper limb and active motion intention and improve the real-time and accuracy of recognition. Based on this, two types of human-machine interaction controllers, which can be called zeroing neural network controllers and noise-suppressing zeroing neural network controllers are designed to establish a safe and comfortable training environment to avoid secondary damage to the affected limb.

In the third paper (Jin et al.), a robust zeroing neural network (RZNN) model for solving dynamic complex matrix equation (DCME) in a noisy-polluted environment is proposed and investigated by introducing a new activation function (NAF). The robustness and convergence of the proposed RZNN model are proven and verified. The proposed RZNN model is applied to manipulate trajectory tracking control, and it completes the trajectory tracking task successfully, which further validates its practical applied prospects.

In the fourth paper (Zhang J. et al.), the adaptive optimal output regulation (AOOR)-based controller is designed for a wheel-legged robot to deal with the possible model uncertainties and disturbances in a data-driven approach. The AOOR-based controller is verified to achieve the optimality of the control performance and stability and shows the effectiveness and high robustness with model uncertainties and external disturbances.

In the fifth paper (Cui et al.), two-point path planning and multi-point path planning methods are proposed for an unmanned aerial vehicle (UAV)-based data collection system. The objective of the research is to maximize the amount of fresh information collected from ground-fixed devices in a complicated forest environment. A chaotic initialization and co-evolutionary algorithm are adopted to solve the two-point path planning issue considering all significant UAV performance and environmental factors.

We appreciate all the authors for their submissions and all the reviewers for their valuable reviews and comments. We hope that this Research Topic will inspire new outcomes for the research community in learning, control, robotic systems, and neural dynamics.

## Author contributions

JZ: writing original draft. LJ: coordinating the Research Topic and writing original draft. TD and MT: reviewing and editing. All authors contributed to the article and approved the submitted version.

